# The arrestin-domain containing protein AdcA is a response element to stress

**DOI:** 10.1186/1478-811X-11-91

**Published:** 2013-11-22

**Authors:** Clémence Habourdin, Gérard Klein, Tsuyoshi Araki, Jeffrey G Williams, Laurence Aubry

**Affiliations:** 1 CEA, iRTSV, Laboratoire Biologie à Grande Echelle, F-38054 Grenoble, France; 2 Université Joseph Fourier, F-38041 Grenoble, France; 3 INSERM, U1038, F-38054 Grenoble, France; 4 College of Life Sciences, Welcome Trust Biocentre, University of Dundee, DD1 5EH, Dundee, United Kingdom; 5 CEA-Grenoble, Institut de Recherche en Technologies et Sciences pour le Vivant, Laboratoire de Biologie à Grande Echelle, Equipe OdyCell, 17 rue des Martyrs, 38054 Grenoble Cedex 09, France

**Keywords:** Arrestin, Stress, STAT transcription factor, Hyperosmolarity, *Dictyostelium*, Phosphorylation

## Abstract

**Background:**

Cell behaviour is tightly determined by sensing and integration of extracellular changes through membrane detectors such as receptors and transporters and activation of downstream signalling cascades. Arrestin proteins act as scaffolds at the plasma membrane and along the endocytic pathway, where they regulate the activity and the fate of some of these detectors. Members of the arrestin clan are widely present from unicellular to metazoa, with roles in signal transduction and metabolism. As a soil amoeba, *Dictyostelium* is frequently confronted with environmental changes likely to compromise survival. Here, we investigated whether the recently described arrestin-related protein AdcA is part of the cell response to stresses.

**Results:**

Our data provide evidence that AdcA responds to a variety of stresses including hyperosmolarity by a transient phosphorylation. Analysis in different mutant backgrounds revealed that AdcA phosphorylation involves pathways other than the DokA and cGMP-dependent osmostress pathways, respectively known to regulate PKA and STATc, key actors in the cellular response to conditions of hyperosmolarity. Interestingly, however, both AdcA and STATc are sensitive to changes in the F-actin polymerization status, suggesting a common primary sensor/trigger and linking the stress-sensitive kinase responsive for AdcA phosphorylation to the actin cytoskeleton. We also show that STATc-dependent transcriptional activity is involved for the timely dephosphorylation of AdcA in cells under stress.

**Conclusion:**

Under osmotic stress, AdcA undergoes a phosphorylation-dephosphorylation cycle involving a stress-sensitive kinase and the transcription regulator STATc. This transient post-transcriptional modification may allow a regulation of AdcA function possibly to optimize the cellular stress response.

## Background

Cells are continuously subjected to environmental cues that determine their behaviour in terms of motility, adhesion, growth and differentiation. Their capacity to sense and respond to external stimuli largely relies on membrane proteins including receptors, adhesion molecules, channels and transporters that function as sensors and signal transducers. In the cytoplasm, adaptor proteins from the arrestin clan play key roles in the signal relay, acting downstream of transmembrane spanning proteins at the plasma membrane or/and along the endocytic pathway. A well-characterized system is the coupling of β-arrestins to ligand-activated G-protein coupled receptors (GPCRs) allowing their temporal desensitization, their trafficking to specific endocytic compartments and the activation of secondary signalling cascades [1-5]. Over the past few years, a family of arrestin-related proteins have been identified in mammals (Arrdcs) as well as in lower organisms such as fungi (ARTs) and amoebae (Adcs) [6-9]. Recent data indicated that ART adaptor proteins regulate the fate of plasma membrane permeases through a conserved ubiquitination-dependent pathway in response to changes in nutritive or pH conditions. ARTs allow the recruitment of the Rsp5 ubiquitin ligase to the vicinity of membrane targets, leading to their ubiquitination and subsequent degradation [8,10-13]. The functions of Arrdcs are still under investigation but recent studies indicate that these proteins could similarly behave as ubiquitin ligase adaptors allowing the regulated ubiquitination of plasma membrane targets and their ensuing downregulation [14,15].

The social amoeba *Dictyostelium* harbours 6 arrestin-domain containing proteins (AdcA to F). Their expression is highly regulated during the developmental cycle but the role of the Adcs at the different stages of the developmental program is completely enigmatic. In contrast to canonical visual and β-arrestins, the *Dictyostelium* Adcs contain additional functional domains besides the arrestin core, including lipid binding- and protein-protein interaction domains [7]. The AdcA protein is extended on both sides of the arrestin core by an N-terminal histidine-rich domain and a C-terminal FYVE domain. This latter domain contributes to the constitutive association of AdcA to the endocytic pathway [16]. The AdcA protein is most highly expressed during the unicellular growth phase of *Dictyostelium*. At this stage, cells are faced with a broad range of environmental changes, ranging from hypo- or hyper-osmotic conditions to nutrient deprivation and their survival in such conditions relies on multiple and complex strategies. Hyperosmotic challenge activates an intricate transcriptionally regulated osmostress response [17].

Our data presented here provide evidence that AdcA undergoes a massive phosphorylation in response to a variety of stresses, including hypertonicity, and that AdcA dephosphorylation is partly dependent on a STATc pathway and correlates with cell recovery, raising the question of a possible role of AdcA in stress response.

## Results

### AdcA is multiphosphorylated in response to sorbitol-induced hyperosmotic stress

**Figure 1:**
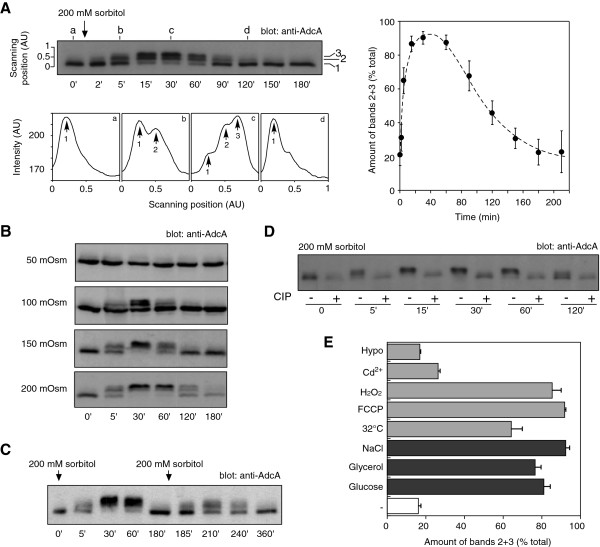
**AdcA is phosphorylated in response to various stresses.** Parental KAx-3 cells in suspension in nutritive medium were subjected to different stress conditions. Protein samples were prepared as described in Materials and Methods and AdcA electrophoretic profile was followed by Western blot with anti-AdcA antibodies. **A**. Effect of sorbitol on AdcA electrophoretic mobility. Cells were treated with 200 mM sorbitol. The bottom panel represents the pixel intensities along the scanning lines a, b, c and d on the top panel. Bands 1, 2 and 3 were quantified using ImageJ software and the signal intensity of bands 2 + 3 was expressed as a percentage of the total and plotted as a function of time. The data shown correspond to the mean ± s.d of 4 experiments. **B**. Effect of increasing concentration of sorbitol on AdcA. Cells were subjected to concentrations of sorbitol ranging from 50 to 200 mM and the consequences on AdcA migration were analysed by Western blot. **C**. AdcA responds to successive sorbitol shocks. Cells were stimulated with 200 mM sorbitol at t = 0 and again at t = 180 min. Samples were collected at indicated times to analyse AdcA response. **D**. Treatment of AdcA with the CIP phosphatase abolishes its mobility shift. Protein samples obtained from cells subjected to 200 mM sorbitol were treated with CIP for 30 min at 37°C (+). Controls (−) were subjected to the exact same procedure but received no enzyme. **E**. AdcA responds to other stresses. Cells were faced with different stress conditions. The amount of AdcA as bands 2 + 3 at t = 30 min was represented as a percentage of the total amount of AdcA. The data are shown as the mean ± s.e.m of 3 independent experiments for each stress and 8 for the control (−) experiments.

**Figure 2:**
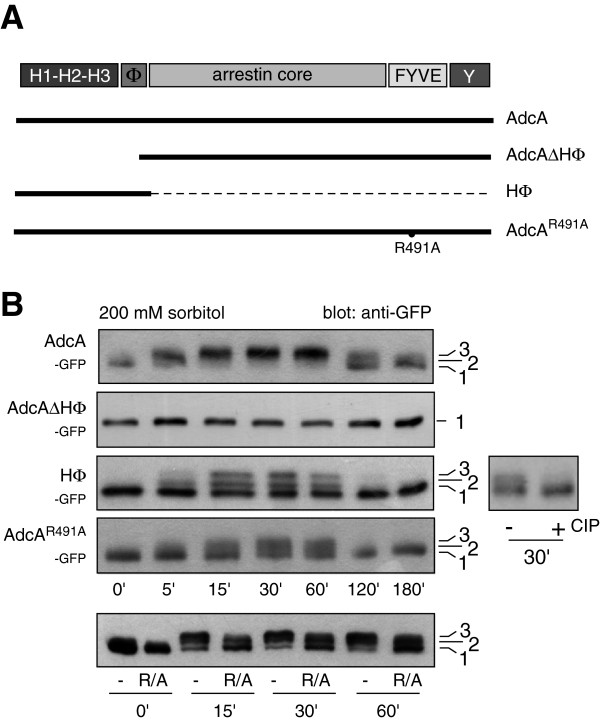
**AdcA is phosphorylated on its N-terminal domain. A**. Schematic representation of mutants of AdcA. AdcA harbors different domains: a triplicated histidine-rich region (H1-H2-H3), a hydrophobic sequence (Φ), an arrestin core, a FYVE domain and a tyrosine-rich region (Y). Mutants of GFP-tagged AdcA carrying a point-mutation in the FYVE domain (AdcA^R491A^) preventing endocytic targeting or truncations (HΦ, AdcAΔHΦ) are shown. **B**. Effect of sorbitol on the migration pattern of AdcA mutants. Cells disrupted for *adcA* and expressing mutant forms of AdcA were treated with 200 mM sorbitol (t = 0). The impact of the hyperosmotic stress on the electrophoretic migration of the different AdcA mutants was analysed by Western blot using anti-GFP antibodies. To confirm that the shift observed with the HΦ protein was due to phosphorylation, the protein sample was treated with the alkaline phosphatase CIP. On the bottom panel, the samples from *adcA* null cells overexpressing AdcA_GFP_ or AdcA^R491A^_GFP_ were loaded side by side to facilitate comparison.

In preliminary experiments, we observed that changes of external medium (12 mM Na,K-Pi buffer vs HL5 axenic medium) affected the electrophoretic pattern of the AdcA protein. We therefore examined the impact of various cellular stresses especially osmotic stress to determine the signal(s) responsible for this modification. In our assays, exponentially growing cells were centrifuged, resuspended in fresh nutritive medium and left to recover in shaking suspension for at least 1 h 30 prior to stress application. This protocol avoided, thereafter, any possible stress due to centrifugation (transient anaerobiosis, mechanical constrains, temperature shift) and nutrient depletion. In these conditions, in KAx-3 cells, AdcA was detected as a major higher mobility form and occasionally a minor slower form (Figure 1A, bands 1 and 2). Addition of 200 mM sorbitol to the medium led to a massive and transient modification of the protein. Within minutes after sorbitol addition, the electrophoretic mobility of AdcA gradually decreased with the accumulation of band 2 and the appearance of a slow migrating third band (Figure 1A, band 3). A similar mobility change was observed with the C-terminally tagged versions of AdcA, AdcA_GFP_ (Figure 2) and AdcA_myc_ (not shown) indicating that the tagged and overexpressed proteins behave as the endogenous AdcA. Maximal amount of band 3 occurred past 30 min post stress application (Figure 1A, right panel). Within the next hour, bands 2 and 3 of AdcA progressively disappeared in favour of the fast migrating band, leading to a migration pattern at 120 min after stimulation comparable to that observed in untreated cells. The extent and duration of each phase was dose-dependent as illustrated on Figure 1B on KAx-3 subjected to increasing concentrations of sorbitol (50 to 200 mM). Treatment of the cells with a second sorbitol-mediated osmotic challenge 180 min after the first shock resulted in a new round of AdcA response (yet not as efficient), indicating that the cells were able to reactivate the pathway(s) governing AdcA response and therefore, at least partially, recover from stress (Figure 1C).

To identify the type of modification(s) responsible for AdcA shift, protein samples were treated with calf intestine phosphatase (CIP) to test the possibility of phosphorylation. As shown in Figure 1D, CIP treatment of protein samples from sorbitol-stressed cells harbouring bands 2 and 3 restored the band 1-migration pattern of unstimulated cells (fast migrating species) while buffer alone had no effect. Similar results were obtained with λ-phosphatase (not shown). These data strongly suggest that AdcA is modified by multiple phosphorylations when subjected to hyperosmotic stress. The dephosphorylation protocol we used includes a step of sample concentration by precipitation in trichloroacetic acid. Such conditions are unlikely to maintain the acid-labile phosphorylation of residues such as histidine and aspartate. Because the use of several phospho-tyrosine specific antibodies failed to detect any phospho-AdcA in an AdcA immunoprecipitate, we currently favor the hypothesis of a phosphorylation on serine/threonine residues.

### AdcA is modified in response to various stresses

To determine whether AdcA response to sorbitol reflected a general sensitivity to osmostresses, other hyperosmotic agents were tested. As shown on Figure 1E, 200 mOsm concentrations of glucose, glycerol or NaCl were all capable of triggering AdcA phosphorylation. As observed for sorbitol, the effect of NaCl is similarly dose-dependent (not shown). Conversely, a hyposmotic shock obtained by shifting the cells from axenic medium to 5-fold diluted medium (130 mOsm to 26 mOsm) did not affect AdcA behaviour (Figure 1E, Hypo). To evaluate whether AdcA phosphorylation is part of a general stress response, cells were subjected to non-osmotically active stresses. AdcA responded equally well to heat shock (32°C), mitochondrial uncoupler (10 μM FCCP), oxidative stress (2 mM H_2_O_2_) but only weakly to a 50 μM Cd^2+^ treatment, indicating that AdcA is sensitive to a large variety of cell stresses (Figure 1E). The pathways activated in response to heat shock, energetic and oxidative stresses have not been characterized in details but given our results, it is possible that they all share an effector, common to the osmostress cascade leading to AdcA phosphorylation.

### AdcA is phosphorylated on the N-terminal HΦ domain

Besides its central arrestin core, the adaptor protein AdcA contains a PI(3)P-binding FYVE domain in the C-terminal part and a histidine-rich triplicated region that extends the protein N-terminally. To localize the site(s) of phosphorylation, the effect of 200 mM sorbitol was measured on truncated forms of AdcA, AdcAΔHΦ_GFP_ and the HΦ_GFP_ expressed in both a KAx-3 and *adcA* null background to exclude a possible interference of the endogenous AdcA (Figure 2A). The construct AdcA_GFP_ was used as a control. A mobility shift induced by sorbitol with the appearance of two slower migrating forms and reversed by CIP treatment was observed for the HΦ_GFP_ construct in both cell backgrounds but was completely lost in AdcAΔHΦ_GFP_, indicating that the N-terminal domain of AdcA contains the phosphorylation sites critical for the shift and the appropriate context for the recruitment of the required kinase and phosphatase (Figure 2B, not shown for the KAx-3 background). Nevertheless, even though the kinetics of phosphorylation of the HΦ_GFP_ protein is the same as that of endogenous AdcA and AdcA_GFP_, the extent of phosphorylation is different with roughly 50 ± 4% (n = 3) of total HΦ_GFP_ in the bands 2 + 3 at 30 min post-shock versus 81 ± 8% (n = 3) for the full-length AdcA_GFP_ protein. This difference could possibly be attributed to the higher level of expression of HΦ_GFP_ and a limiting level of kinase activity. Alternatively, if the subcellular localisation of HΦ_GFP_ were distinct from that of AdcA, this could also interfere with the phosphorylation efficiency. Indeed, while AdcA is massively associated with the endocytic apparatus, the HΦ construct is mostly found in the cytosol [16]. Another mutant of AdcA, AdcA^R491A^_GFP_, that is unable to bind PI(3)P, fails to localize to the endocytic compartments [16]. Similarly to HΦ_GFP_, AdcA^R491A^_GFP_ phosphorylation is partial, despite an expression level comparable to that of AdcA_GFP_, supporting a correlation between the endocytic localization and the phosphorylation level of the protein rather than a phosphorylation level mirroring the expression level of the constructs (Figure 2A, B).

The HΦ domain of AdcA contains 31 serines/threonines (and 2 tyrosine residues), precluding a systematic point-mutation approach to test their relative contribution to the phosphorylation pattern. Attempts to map the phosphorylated site(s) by mass spectrometry on the immunoprecipitated HΦ_GFP_ protein were so far unsuccessful.

### Vegetative cells and starved cells confronted by hypertonicity share common features

In nutritive conditions, *Dictyostelium* cells multiply actively as single cells (vegetative state) and nutrient depletion triggers a developmental program leading to a multicellular structure containing starvation-resistant spores. In *Dictyostelium*, the consequences of a hyperosmotic stress have been well characterized on cells starved for a 1–4 h period of time and several effectors of the osmoresponse have been identified [18-26]. Hyperosmotic conditions trigger a variety of responses, such as cell shrinkage, cytoskeleton rearrangement and actin/myosin phosphorylation. High osmolarity responses include a rapid and transient accumulation of intracellular cAMP and of intracellular cGMP that functions, in parallel to Ca^2+^-based pathways, to activate the stress-activated transcription factor STATc [19,21,26,27].

**Figure 3:**
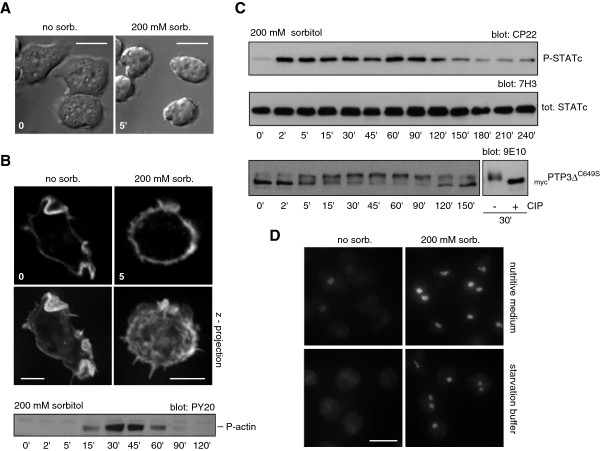
**Responses of vegetative KAx-3 cells to hyperosmolarity.** Cells maintained in culture medium were treated with 200 mM sorbitol and the consequences of hyperosmolarity were followed on different aspects of the cell biology. **A**. The effect on cellular morphology was monitored by time-lapse video microscopy using DIC optics and a 63x objective. The images shown illustrate the morphology of the cells after 5 min of stress. Scale bars: 10 μm. **B**. The distribution of F-actin was analysed using Alexa 594-phalloidin after cell fixation/permeabilisation in 4% PFA, 0.1% Triton X-100. To enhance resolution, optical sections were taken throughout the cell and digitally deconvolved using Axiovision software. One optical section and a z-stack maximal intensity projection are shown. The phosphorylation of actin in response to sorbitol treatment was examined by Western blot on whole cell extracts using the anti-phospho-Tyr antibody (PY20). Scale bars: 5 μm. **C**. Endogenous STATc and _myc_PTP3Δ^C649S^ phosphorylation was analysed using the STATc-specific antibody CP22 and 7H3 or the anti-myc antibody 9E10. **D**. STATc nuclear translocation in response to stress was detected by immunofluorescence using the 7H3 antibody on cells kept in nutritive medium or starved for 4 h in 12 mM Na, K-phosphate buffer, pH 6.2, subjected or not to 200 mM sorbitol for 5 min. Scale bar: 10 μm.

To position AdcA in the osmostress-response signaling network, we first compared the stress response of growing KAx-3 cells to that of starving cells. When treated with 200 mM sorbitol, vegetative cells rounded up and shrunk rapidly, stopped their random motility and detached progressively from the substratum (Figure 3A, Additional file 1: Movie 1). Actin redistributed uniformly around the cell periphery as observed for starving cells, within a few minutes after sorbitol addition (Figure 3B). A transient Tyr-phosphorylation of actin was also detectable 15 min after the shock with a peak around 30 min (Figure 3B). One participant in the osmostress response in starving cells, STATc, was also Tyr^922^ phosphorylated in our conditions within minutes following application of the osmotic stress (Figure 3C). As observed for AdcA and actin, this phosphorylation is transitory as a slow dephosphorylation proceeded past 90 min post-shock. The osmostress activation of the transcription factor STATc was shown to depend on a dual regulation involving a not yet identified tyrosine-kinase and the tyrosine-phosphatase PTP3, the activity of which is inhibited by phosphorylation on Ser/Thr residues [19,28]. As shown on Figure 3C, the protein _myc_PTP3Δ^C649S^, used as a reporter of PTP3 behaviour [27], was also transiently phosphorylated (as validated by the CIP effect), thereby likely contributing to STATc activation. STATc phosphorylation was accompanied by its translocation into the nucleus (Figure 3D). Noteworthy, while STATc is massively cytosolic in early developing cells, we could detect some staining in the nucleus of growing cells suggesting a basal level of activation of the transcription factor in vegetative conditions. This is supported by the low but detectable basal level of phospho-STATc observed in growing cells (Figure 3C).

### Efficient AdcA phosphorylation requires pathways other than the DokA and cGMP-dependent osmostress pathways

**Figure 4:**
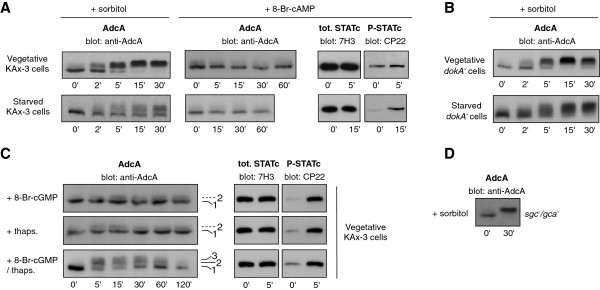
**Effect of various mutants and addition of 8-Br-cAMP, 8-Br-cGMP and thapsigargin on AdcA phosphorylation. A**. AdcA is not phosphorylated in response to 8-Br-cAMP. Vegetative or 4 h-starved cells in KK2 buffer (16.5 mM KH_2_PO_4_, 3.8 mM K_2_HPO_4_, pH 6.2) were treated with 20 mM 8-Br-cAMP or 200 mM sorbitol. **B**. AdcA is not dependent on DokA. *dokA* null cells kept in nutritive medium or starved for 4 h in KK2 buffer were treated with 200 mM sorbitol. **C**. AdcA is partly phosphorylated in response to a combination of 8-Br-cGMP and thapsigargin. Vegetative KAx-3 cells were treated with 20 mM 8-Br-cGMP, 10 μM thapsigargin or a mix of 8-Br-cGMP/thapsigargin. **D**. The response of AdcA is not affected in *sgc*/*gca* double null mutant. The *sgc/gca* null strain disrupted for the 2 guanylate cyclases sGC and GCA were subjected to 200 mM sorbitol. In all tested conditions, the response of AdcA was followed by Western blot using anti-AdcA antibodies **(A, B, C and D)**. The phosphorylation of STATc in the same conditions was detected using the 3H7 and CP22 antibodies and was used as a positive control of cell responsiveness **(A and C)**.

We next examined whether some of the secondary messengers described to function in the osmoresponse of starving cells (i.e. Ca^2+^ and cyclic nucleotides) operate to trigger AdcA stress-induced response. In hyperosmotically-stressed starving cells, a peak of cAMP is detected, due to the activation of the histidine kinase DokA and the subsequent transient inhibition of the cytosolic cAMP phosphodiesterase RegA [21,24]. The addition of the membrane-permeable analogue 8-Br-cAMP, used to mimic an increase in the cAMP cytosolic concentration, failed to activate AdcA in growing cells (Figure 4A). As such treatment only triggered a low level of STATc phosphorylation (used as a control), the effect of 8-Br-cAMP was also tested on 4 h starved cells, conditions known to induce a substantial response of the transcriptional factor ([18], Figure 4A). While sorbitol treatment also triggered the phosphorylation of AdcA in starved cells (yet not as efficiently as in vegetative cells), no activation of the AdcA response was observed with 8-Br-cAMP (Figure 4A). In agreement with these data, the *dokA* null mutation maintained the sorbitol-inducible phosphorylation of AdcA (Figure 4B). DokA being poorly expressed in vegetative cells, the experiments were also conducted on starved cells showing that the absence of *dokA* did not alter AdcA phosphorylation profile (Figure 4B). A DokA-cAMP pathway is therefore unlikely to participate to AdcA phosphorylation.

We next tested the possible contribution of cGMP- and calcium-dependent pathways. While treatment of cells with 8-Br-cGMP efficiently activated STATc in vegetative cells, its effect on AdcA was very weak (Figure 4C). *Dictyostelium* encodes two characterized guanylate cyclases, the soluble hyperosmotic stress-inducible protein sGC and the membrane-associated GCA protein [23]. The deletion of *gca/sgc* has been shown to result in a complete loss of detectable guanylate cyclase activity in 1 h-starved cells subjected to 300 mOsm challenge. Disruption of both *gca* and *sgc* (*gca*^*-*^*/sgc*^*-*^*)* did not interfere with the full phosphorylation of AdcA in response to sorbitol (Figure 4D). In parallel, thapsigargin was used to induce an elevation of intracytosolic calcium. Again, this treatment caused an accumulation of phosphorylated STATc but barely induced a response from AdcA (Figure 4C). However, the addition of both thapsigargin and 8-Br-cGMP together was able to produce the appearance of band 3 (Figure 4C). The response was partial compared to the sorbitol effect, suggesting that these conditions did not completely mimic those generated by sorbitol. The doses used and their simultaneous addition may not be the most effective combination to trigger AdcA phosphorylation. An alternative hypothesis is that, in conditions of hyperosmotic stress, cGMP and Ca^2+^- dependent pathways function in parallel to additional pathways to elicit a full AdcA response.

### AdcA phosphorylation is induced by a destabilization of the actin network

**Figure 5:**
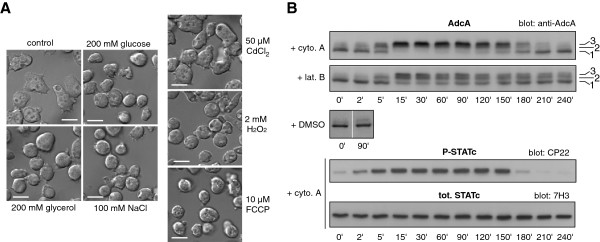
**Involvement of the actin cytoskeleton in AdcA response. A**. Morphological changes induced by various stresses**.** Cells in nutritive medium were let to adhere for 1 h on glass coverslips and were treated with 200 mOsm glucose, glycerol or NaCl, 2 mM H_2_O_2_, 10 μM FCCP or 50 μM Cd^2+^. Cells were analysed 30 min after treatment by DIC microscopy with a 63x objective. All stresses except 50 μM Cd^2+^ led to a rounding up of the cells. Scale bar: 10 μm. **B**. Actin depolymerisation induces AdcA phosphorylation. KAx-3 cells were subjected to 10 μM cytochalasin A or 5 μM latrunculin B (addition at t = 0) and the impact on AdcA and STATc was followed as described above. The control cells received DMSO alone.

All the treatments driving AdcA phosphorylation led to a rounding up of the cells, likely involving a reorganisation of the actin cytoskeleton (Figure 5A). In the case of hyperosmotic stress, a protective cortical shell of F-actin is built through a massive restructuration of the actin network (Figure 3A, [25]). This led us to test whether the sole destabilization of the actin network could trigger AdcA phosphorylation. Cells were treated with two structurally and functionally distinct actin cytoskeleton disrupting agents, cytochalasin A (10 μM) or latrunculin B (5 μM). As shown on Figure 5B, these two drugs led to the activation of STATc as previously described by Araki and Williams on starved cells [29]. Cytochalasin A also induced a robust and rapid response of AdcA with the massive accumulation of band 3, comparable to that obtained in sorbitol treated cells. Latrunculin B (Figure 5B) as well as latrunculin A (not shown) were also able to trigger AdcA phosphorylation, yet not as efficiently. Thus, actin depolymerisation is sufficient to elicit AdcA phosphorylation. Both drug families induced a delay in the dephosphorylation of AdcA, a full dephosphorylation of AdcA being eventually obtained only with cytochalasin A. This difference between the impacts of the drugs may be due to their distinct mode of action and the consequences of the chemicals on the cell physiology. *Dictyostelium* cells may be able to expel cytochalasin A through multidrug resistance pumps of the plasma membrane, allowing the cell to restore the basal parameters including a dephosphorylated AdcA.

### AdcA phosphorylation is not dependent on ERK proteins, PKA or GbpC

**Figure 6:**
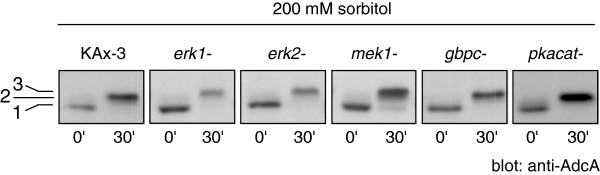
**AdcA phosphorylation is maintained in various kinase mutant strains.** Cells disrupted for *erk1*, *erk2*, *mek1*, *gbpc* and *pkacat* were used to test the impact of 200 mM sorbitol on AdcA in these mutant backgrounds. Samples were harvested prior and 30 min post sorbitol addition and AdcA response was followed by Western blot.

To determine the kinase(s) responsible for AdcA phosphorylation, we tested AdcA response to sorbitol in strains disrupted in candidate kinases. As expected from the absence of AdcA response upon 8-Br-cAMP addition, a deletion mutant in the PKA catalytic subunit (*pkacat*) was still responsive to sorbitol treatment, excluding a role for this Ser/Thr kinase (Figure 6). The small effect of 8-Br-cGMP prompted us to test the cGMP-binding kinase GbpC, an intermediate in the osmostress signaling cascade leading to STATc phosphorylation. Disruption of *gbpC* did not alter AdcA phosphorylation kinetics (Figure 6).

In yeast and mammals, MAP kinases are key actors in the signaling pathways activated in conditions of hyperosmotic stress. In *Dictyostelium*, the involvement of the two MAP kinases Erk1 and Erk2 in the osmostress response has not been addressed in detail but Na and colleagues [17] suggested the implication of a MAPK dependent pathway. Interestingly, the MAP kinase kinase MEK1 is hyperphosphorylated in developing cells subjected to high osmolarity conditions, with kinetics similar to that of STATc [30]. Therefore, the phosphorylation status of AdcA was analysed in *erk1*, *erk2* and *mek1* null mutants (Figure 6). None of the mutations affected AdcA phosphorylation, an observation not in favor of a role for these kinases in the AdcA response.

### AdcA dephosphorylation is under the partial control of STATc

**Figure 7:**
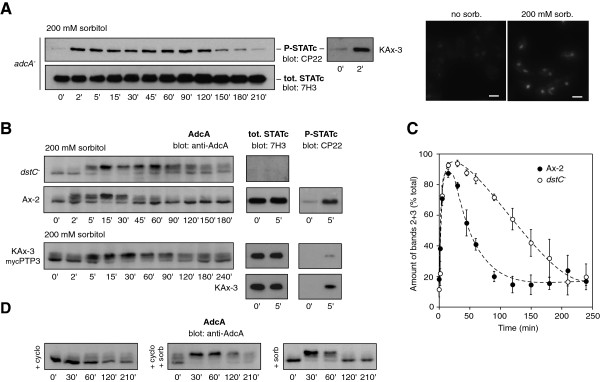
**STATc deletion delays AdcA dephosphorylation. A**. Disruption of *adcA* has no effect on STATc response in conditions of hyperosmolarity. *adcA* null cells were treated with 200 mM sorbitol (t = 0). STATc phosphorylation and nuclear translocation were examined by Western blot (7H3 and CP22 antibodies) and immunofluorescence (7H3 antibody) respectively. Scale bar: 10 μm. **B and C**. STATc disruption leads to a persistence of AdcA phosphorylated status. The STATc knock-out strain (*dstC*^*-*^), Ax-2 cells (*dstC*^*-*^ parent) and KAx-3 cells overexpressing _myc_PTP3 were subjected to 200 mM sorbitol at t = 0 and AdcA phosphorylation was followed by Western blot. The right panels illustrate the level of activation of STATc in these different strains in response to sorbitol **(B)**. The signal intensity of bands 2 + 3 was measured using ImageJ software, expressed as a percentage of the total signal intensity (bands 1 + 2 + 3) and plotted as a function of time. The normalized data are shown for the *dstC*^*-*^ and Ax-2 cells as the mean ± s.e.m. (n = 3) **(C)**. **D**. Cycloheximide mimics the effect of STATc disruption. KAx-3 cells were treated with 2 mM cycloheximide alone or 30 min prior to treatment with 200 mM sorbitol and AdcA electrophoretic profile was followed by Western blot.

Given that both AdcA and STATc responded to similar stresses (our present data; [18]), we examined the possibility of any functional link between these two targets of the osmostress. In a previous work, an *adcA* null strain was generated by interruption of the coding sequence by a blasticidine resistance cassette [16]. The deletion of *adcA* has no noticeable effect on growth and endocytosis (as measured by uptake of FITC-dextran or latex beads) (D. Guetta, GK and LA, unpublished observations). We first tested the impact of this disruption on STATc activation. As shown in Figure 7A, the absence of *adcA* did not affect STATc’s tyrosine phosphorylation/dephosphorylation when compared to KAx-3 or its translocation to the nucleus, indicating that AdcA is not an essential intermediate in the pathway controlling STATc response.

Conversely, the *dstC* deletion (*statc* null strain, Ax-2 background) affected the phosphorylation status of AdcA. In the two *dstC*^*-*^ and Ax-2 strains, the phosphorylation peak was similarly attained around 30 min post sorbitol stimulation but then, in the *dstC* mutant, the protein remained phosphorylated for an extended period of time as evidenced by the massive amount of band 3 still present at 150 min (42 ± 9% in *dstC*^-^*vs* 14 ± 6% in Ax-2) (Figure 7B and C). To further investigate a role for STATc in AdcA dephosphorylation, we repeated the same experiment in KAx-3 cells expressing various forms of PTP3 [31]. The tyrosine phosphatase PTP3 is responsible for the dephosphorylation of STATc [28]. Accordingly, the constitutive overexpression of PTP3 leads to a reduced STATc activation while overexpression of a dominant negative form of PTP3 mutated in the catalytic cysteine residue (_myc_PTP3Δ^C649S^) leads to a constitutively active STATc [28]. As shown on Figure 7B, exposing KAx-3 overexpressing _myc_PTP3 to 200 mM sorbitol led to a response of AdcA that resembles that observed in the *dstC* null strain with a persistence of AdcA phosphorylation, several hours after shock (Figure 7B). Such result suggests a role for STATc transcriptional activity in AdcA dephosphorylation. This was further supported by experiments using cycloheximide, a general protein synthesis inhibitor. While 2 mM cycloheximide by itself had only a slight effect on AdcA (possibly due to the generation of a situation of stress after long exposure to the inhibitor), pre-treatment of KAx-3 cells with cycloheximide, for 30 min prior sorbitol addition significantly slowed down AdcA dephosphorylation with persistence of band 3 for the next 180 min (Figure 7D), suggesting the requirement of protein neosynthesis for dephosphorylation to occur. In KAx-3 cells overexpressing _myc_PTP3Δ^C649S^ however, the phosphorylation of AdcA was not noticeably affected despite a constitutive activation of STATc (not shown). Taken together, the data support a functional link between STATc activity and AdcA dephosphorylation. However, while STATc could very well control the expression of a component of the AdcA dephosphorylation pathway, an additional level of regulation possibly acting on the balance between the activity of the AdcA-specific kinase and that of its phosphatase must be invoked to account for the absence of effect of constitutively activated STATc.

### Impact of dstC and adcA disruption on cell resistance to stress

**Figure 8:**
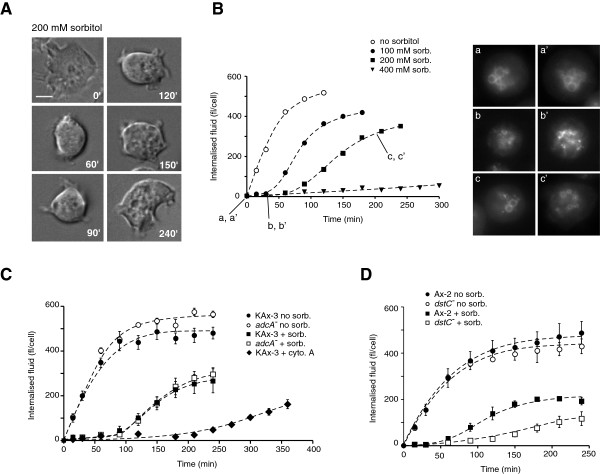
**Cell recovery temporally correlates with AdcA dephosphorylation. A**. KAx-3 cells were let to adhere on a glass coverslip in culture medium for 1 h. Cells were then treated with 200 mM sorbitol in culture medium and observed by time-lapse videomicroscopy using DIC optics. The images illustrate the cells at different stages post sorbitol addition. Scale bar: 5 μm. **B**. KAx-3 in shaking culture received simultaneously 2 mg/ml FITC-dextran and various concentrations of sorbitol (0, 100, 200 and 400 mM). Aliquots were collected as a function of time and treated as described in Methods. The volume of internalized fluid was expressed in fl/cell. Data correspond to a representative experiment. KAx-3 overexpressing AdcA_GFP_ were used in parallel to follow the localisation of AdcA during the stress response. Cells from a shaking culture were removed prior (t = 0, a, a’) and after sorbitol addition (t = 30 min (b,b’) and t = 210 min (c,c’)) and observed directly by fluorescence microscopy. Two representative cells are shown. **C**. Shaking cultures of KAx-3 and *adcA* null cells were treated with 200 mM sorbitol or 10 μM cytochalasin A (only for KAx-3). Adaptation to treatments was monitored by the resumption of the endocytic activity as described in B using FITC-dextran as a fluid-phase marker. Mean ± s.e.m. (n = 3). **D**. Ax2 and *dstC*^*-*^ cells were subjected to 200 mM sorbitol and their endocytic activity was evaluated as described above. Mean ± s.e.m. (n = 3).

We next undertook a characterization of the cell behaviour at the time of AdcA dephosphorylation. When subjected to a stress of 200 mM sorbitol, the cell rounding and actin rearrangement induced within the first minutes after shock were transitory. As illustrated on Figure 8A, cells adapted progressively to their new environment and regained a partly adherent and motile behaviour around 90 min following the onset of osmotic stress. Pintsch and colleagues described that hyperosmolarity rapidly blocked vesicular trafficking and macropinocytosis with a fragmentation of the endocytic pathway [22]. In agreement with that observation, while localized on large size macropinosomes in the absence of stress, AdcA was found associated to small size structures compatible with a partially fragmented endocytic network in sorbitol-treated cells (Figure 8B, [16]). Our data also confirmed the arrest of the endocytic activity as shown by the inhibition of the internalization of the fluid-phase marker FITC-dextran within the first minutes following the addition of 200 mM sorbitol (Figure 8B). However, this arrest is transient. Indeed, past 90 min of stress and in parallel to cell spreading and recovery of motility, KAx-3 cells had resumed some macropinocytic activity (Figure 8B, 200 mM sorb.). Past the adaptation phase, AdcA localization on large size endocytic vesicles was again visible (Figure 8B, c and c’). Lower concentrations of sorbitol (100 mM) allowed a faster cell recovery assessed by endocytosis measurements while higher, yet not lethal, concentrations (400 mM) prohibited adaptation at least during a 5 h time window. In all cases, recovery of the cells temporally correlated with AdcA dephosphorylation (Figure 1A, B and Figure 8B). We reported above that a dephosphorylation of AdcA also occurred in cells maintained in the presence of cytochalasin A. In these conditions, an endocytic activity was eventually restored by 180 min post cytochalasin A addition, possibly due to the pumping of cytochalasin A outside of the cells via MDRs and the subsequent restructuration of a functional actin cytoskeleton. In such cells, the regain of endocytic activity also paralleled AdcA dephosphorylation (Figures 5B, 8C).

We next asked whether *adcA* disruption modified cell resistance to stress and recovery. To test a possible contribution of AdcA to the osmoresponse, parental KAx-3 and *adcA* null cells were first subjected to increasing concentrations of sorbitol (100 to 400 mM) and cell growth was followed on a 96 h period of time. Addition of sorbitol affected cellular growth in a dose-dependent manner but similarly in *adcA* null and control cells suggesting that *adcA* deletion had no significant consequences on survival in hyperosmotic conditions (not shown). Analysis of the *adcA* null cell response in greater detail within the first four hours following a 200 mM sorbitol treatment confirmed that the absence of AdcA has no impact on cell recovery as indicated by the macroscopic morphological changes (not shown) and restoration of endocytosis within the same time frame as for KAx-3 cells (Figure 7C).

Given the impact of the *dstC* null mutation on AdcA dephosphorylation, we analysed the effect of the absence of STATc activity on cell adaptation to stress. Data from Araki and colleagues [18] established that STATc is not essential for stress resistance as assayed by regrowth on *Klebsiella* after 2 h-exposure to 200 mM sorbitol. We tested here, by a less stringent discriminator, the capacity of the *dstC* null cells to restore some endocytic activity within the first hours after the onset of shock. As shown in Figure 8D, while Ax-2 cells regained an endocytic behaviour within 50–60 min post shock, the absence of STATc led to a delay in recovery, comparable to the delay observed in the kinetics of AdcA dephosphorylation.

## Discussion

In the present study, we provide evidence that AdcA, one of the amoebal arrestin-related proteins, is a response element to a number of stresses in growing cells. Using a hyperosmotic shock induced by sorbitol as a paradigm, we showed that the sensing of the shock by vegetative amoebae is accompanied not only by the phosphorylation of the known actors of the osmostress response, the tyrosine phosphatase PTP3 and the transcription factor STATc but also by a multi-phosphorylation of AdcA, most probably on serines/threonines. Adaptation of the cells to the osmostress situation temporally correlates with the dephosphorylation of AdcA.

**Figure 9:**
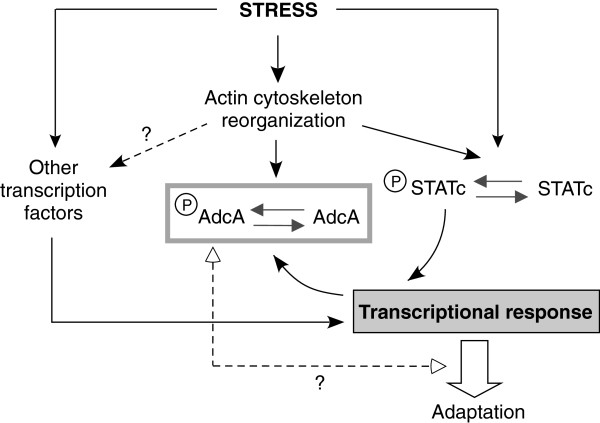
**A model for AdcA in the stress response.** The model proposes that hyperosmolarity, as various other stresses, leads to a reorganization of the cytoskeletal network, and the consequent phosphorylation of response elements including STATc and AdcA, possibly through the activation of a membrane sensor. STATc participates to the cellular transcriptional response triggered by stress. AdcA dephosphorylation is in part dependent on STATc activity and correlates with recovery of the cells from stress. Whether AdcA phosphorylation allows the activation or the inhibition of AdcA is not known, but this phosphorylation or the following dephosphorylation may be required for a full cell recovery.

Given that hyperosmolarity evokes AdcA phosphorylation, we tried to position AdcA relatively to known mediators of the osmoresponse. In response to the osmotic shock, vegetative cells like starving cells shrink rapidly and modify their cortical actin. In starving cells, distinct pathways have been described that contribute to the cell response in conditions of high osmolarity: i) cGMP- and Ca^2+^-dependent pathways involved in the cytoskeletal remodelling and the activation of the transcription factor STATc [19,26,32] and ii) a two-component system dependent pathway including the histidine kinase DokA, the intermediate histidine phosphotransfer protein RdeA and the cAMP phosphodiesterase RegA [21,24]. Whether a signaling cascade involving MAPKs is also active as described in yeast and mammals is still unclear but has been suggested by Na and colleagues [17]. Using known inducers of these signaling cascades or null mutants in some of their respective effectors, our data indicate that AdcA phosphorylation results from the integration of signals arising from multiple pathways including cGMP and Ca^2+^-dependent ones. Interestingly, remodelling of the actin cytoskeleton by F-actin disrupting drugs is an efficient inducer of AdcA phosphorylation. Though AdcA and STATc responses are both elicited by common stresses including F-actin destabilization (this work, [18,29]), an early branching off of the pathways leading to the phosphorylation of AdcA and STATc is therefore likely to occur (Figure 9). In the case of STATc, the cGMP effect is mediated by the Roco kinase GbpC, a regulator of the cytoskeletal network. This pathway is not essential for AdcA response. The partial effect of the concomitant increase of cGMP and Ca^2+^ on AdcA phosphorylation therefore needs to imply other targets for the secondary messenger cGMP as well as other intermediates, possibly involved in the remodelling of the actin network.

The identification of the kinase responsible for AdcA’s phosphorylation would clearly help positioning AdcA in the signaling cascade. In addition to GbpC, our data disqualified PKA, Erk1, Erk2 and MEK1 as key kinases in AdcA phosphorylation. The amoebal kinome includes 255 kinases, with 155 putative Ser/Thr-directed kinases [33]. As AdcA is associated with the PI(3)P-enriched endosomal membrane, we could expect its kinase to be membrane-associated, all the more as soluble mutants of AdcA, e.g. the HΦ domain or the AdcA^R491A^ construct, were less efficiently phosphorylated than AdcA. Alternatively, the hypothesis that folding of the soluble domains of AdcA and of AdcA^R491A^ are different enough from that of AdcA to decrease the affinity for the kinase would perfectly accommodate a soluble kinase. It is thus impossible at this stage to make an informed guess as to which kinase is responsible for AdcA’s phosphorylation. In mild conditions of stress (100–200 mM sorbitol), AdcA phosphorylation is transient and its dephosphorylation is partly dependent on STATc. Given the transcriptional activity of STATc, a simple hypothesis is that STATc regulates the expression of a mediator of AdcA dephosphorylation such as the phosphatase itself or a regulator of the enzyme. Na and colleagues established by means of DNA microarray profiling the transcriptional profile of vegetative cells challenged with high sorbitol, and, among the differentially regulated genes, those that were dependent on the presence of STATc [17]. The list of osmostress-induced STATc-regulated genes contains only one protein phosphatase, DDB_G0274153. DDB_G0274153 encodes a putative serine-threonine protein phosphatase 2C expressed in vegetative conditions. This phosphatase may regulate AdcA phosphorylation and will receive a particular attention. However, AdcA is phosphorylated on multiple sites, STATc inactive mutants permit a partial dephosphorylation of AdcA and the constitutive activation of STATc is mostly silent while a reduction of the extent of AdcA phosphorylation would be expected. The regulation of AdcA response is thus likely to be more complex and involve parallel and complementary pathways in both the phosphorylation and the dephosphorylation steps.

In higher eukaryotes, β-arrestins are recruited to GPCRs stimulated by ligand binding. This leads to the uncoupling of the receptors from their associated G proteins, the arrest of downstream signaling and the internalization of some of these GPCRs in clathrin-coated vesicles through the interaction of the arrestins with components of the endocytic machinery and the activation of signaling cascades from the endosomal platform [4,34]. In yeast, more recent data have established that α-arrestin-dependent pathways are activated in response to stresses and changes in nutritive conditions or ambient pH, triggering the trafficking of plasma membrane permeases. Targets include the general amino acid permease Gap1 (and the α-arrestins Bul1, Bul2), the arginine transporter Can1 (ART1), the lactate permease Jen1 (Rod1), the manganese transporter Smf1 (ART2, ART8) or the 7-transmembrane pH sensor Rim21 (ART9/Rim8) [8-13,35]. Arrestins and arrestin-like proteins are regulated by phosphorylation. In the absence of ligand stimulation, β-arrestins are cytosolic in an inactive, phosphorylated oligomeric form and their recruitment to a phosphorylated GPCR requires their dephosphorylation [36-39]. When the nutrient source is restricted, the phosphorylated forms of α-arrestins are maintained in a complex with the scaffolding protein 14-3-3 [10,12]. The provision of nutrients activates their dephosphorylation, allowing them to target the E3 ubiquitin ligase Rsp5 on specific cargoes, thereby promoting cargo downregulation [8,10,12]. How does the phosphorylation/dephosphorylation response of AdcA to stress compare to that experienced by α-and β-arrestins? We have shown that hyperosmotic stresses, as well as oxidative or heat shock conditions trigger a massive phosphorylation of the pool of AdcA. Whether this phosphorylation leads to the activation or the inactivation of AdcA is not yet established as the exact role of AdcA is currently unknown and none of AdcA membrane targets has been identified. The limited consequences of *adcA* deletion on cell response to hyperosmolarity would rather argue against a role for AdcA as an actor of the osmostress response. However, to provide a definite conclusion on the significance of AdcA phosphorylation in the stress context, identification of the phospho-sites and the subsequent construction of phospho-mimicking and non-phosphorylable mutants of the protein will be essential. We showed that, in various contexts of stress affecting cell morphology and endocytic activity, AdcA response consistently correlates with cell behaviour. AdcA being a protagonist of the endocytic pathway, its phosphorylation could modify its function and interfere with the regulation and fate of some key membrane targets (e.g. ion transporters) involved in the stress response. While the absence of AdcA may be silent, its phosphorylated and/or dephosphorylated form could be necessary intermediates for an optimal response and reaching of a novel homeostasis (Figure 9) if one of these forms was to act as an inhibitor in the response.

The responsiveness of AdcA to various types of stress raises the question of the nature of the cellular trigger common to all the stresses, and able to elicit AdcA phosphorylation. In eukaryotes, defects of the cytoskeleton are one of the major damages observed in response to stress conditions. In *Dictyostelium*, two observations support a role for the cytoskeleton as a “stress transducer”, common to all stress conditions: (i) stressors described to trigger AdcA phosphorylation all alter the cellular morphology and (ii) cytochalasin A as well as latrunculins are able to induce AdcA response in the absence of any other stressor. Given the intricate network of interactions between cytoskeletal elements and transmembrane or membrane-associated proteins, remodelling of the actin cytoskeleton could directly contribute to the activation/inhibition of signaling cascades and the transmission of signals to downstream stress-response effectors, among which AdcA kinase.

## Conclusions

In the telluric amoebae *Dictyostelium*, hypertonicity triggers immediate morphological changes with remodelling of the cytoskeleton and activation of a transcriptional response affecting the expression of hundreds genes. Our data identified a novel target of the osmostress response, the arrestin-related protein AdcA, the regulation of which by a phosphorylation/dephosphorylation cycle might allow an optimal response of cells under stress.

## Methods

### Strains and cell culture conditions

Except when mentioned, the experiments were conducted on the *Dictyostelium discoideum* parental strain KAx-3. The null cells *gbpC*^*-*^ (DBS0302680)*, mek1*^*-*^ (DBS0236541), *pkacat*^*-*^ (DBS0236783) and *gca*^*-*^*/sgc*^*-*^ (DBS0236000) were obtained from the Dicty Stock Center. The *erk1*^*-*^*and erk2*^*-*^ null cells were kindly provided by J. Hadwiger and the *dokA*^*-*^ mutant was obtained from S. Schuster. The *adcA*^*-*^ (parent KAx-3, bs^R^) and strains overexpressing AdcA constructs (G418^R^) (KAx-3/AdcA_myc_, KAx-3/AdcA_GFP_, KAx-3/AdcAΔHΦ_GFP_, KAx-3/HΦ_GFP_, KAx-3/AdcA^R491A^_GFP_, *adcA*^*-*^*/*AdcA_GFP_, *adcA*^*-*^*/*AdcAΔHΦ_GFP_, *adcA*^*-*^*/*HΦ_GFP_, *adcA*^*-*^*/*AdcA^R491A^_GFP_) were described previously [16]. The *dstC*^-^ (parent Ax-2, hyg^R^) was described earlier [18]. KAx-3/_myc_PTP3 and KAx-3/_myc_PTP3Δ^C649S^ were obtained by electroporation in KAx-3 cells of the _myc_PTP3 and _myc_PTP3Δ^C649S^ overexpressing constructs (G418^R^) derived from the work of the Firtel laboratory [27]. All *Dictyostelium* strains were grown in Petri dishes or in shaking culture at 21°C in the presence of appropriate antibiotics in maltose-containing axenic medium [40] except for Ax-2 and *dstC*^-^ that were grown in HL5 medium (Formedium, UK) supplemented with 14 g/l glucose.

### Stress response activation

Cells were harvested in log-phase from shaking cultures and resuspended in nutritive medium at 10^7^ cells/ml. After 1 h 30-recovery in shaking suspension at 21°C, cells were subjected to different stress conditions: heat shock at 32°C or sorbitol, NaCl, glucose, glycerol, carbonyl cyanide 4-(trifluoromethoxy)phenylhydrazone (FCCP, Sigma-Aldrich, Saint Quentin Fallavier, France), CdCl_2_ and H_2_O_2_ (Sigma-Aldrich) at concentrations indicated in the text. For treatment with sorbitol, glucose, NaCl and glycerol, 10× stocks diluted in appropriate medium depending on the experiment (nutritive medium or phosphate buffer) were used. For heat shock experiments, cells were transferred to a 32°C shaking water bath. Samples of 200 μl were removed just prior (t0) and during stress application as a function of time. Cells were rapidly centrifuged, resuspended in denaturing buffer (60 mM Tris–HCl, pH 6.8, 100 mM DTT, 6% glycerol, 2% SDS plus traces of bromophenol blue) and boiled for 3 min at 94°C. To test the contribution of the cytoskeleton, cells were treated with 5 μM latrunculin B (Sigma-Aldrich) or 10 μM cytochalasin A (Sigma-Aldrich) and DMSO was used as control. 8-Br-cAMP (20 mM) (Enzo Life Sciences, Villeurbanne, France), 8-Br-cGMP (20 mM) (Enzo) and thapsigargin (10 μM) (Alomone Labs, Jerusalem, Israel) were used to test the impact of an increase in cAMP, cGMP and an elevation of intracellular calcium respectively. For experiments performed on adherent cells, stress was applied after 1 h 30 of recovery, by removal of the nutritive medium and addition of fresh medium containing the appropriate concentration of stressor.

### Western blot

Protein samples corresponding to 2 × 10^5^ cells were separated by SDS-PAGE and transferred on PVDF membranes. AdcA was detected using the guinea-pig anti-AdcA antibody (COB25) [16] in TBS-0.05% Tween-20 containing 1% BSA. STATc phosphorylation status was followed using the monoclonal phospho-specific (CP22) or non phospho-specific (7H3) antibodies as described [41]. The anti-phosphoTyr antibody PY20 used to detect Tyr53-phosphorylated actin was from SantaCruz (CliniSciences, Nanterre, France). The anti-GFP (clones 7.1 and 13.1) and the anti-myc 9E10 antibodies were purchased from Roche Diagnostics (Meylan, France). Line scans and blot analyses were done with ImageJ software.

### Fluid-phase endocytosis assay

Cells were resuspended at the concentration of 10^7^ cells/ml in axenic medium. FITC-dextran (2 mg/ml final) and sorbitol (100, 200 and 400 mM final) or cytochalasin A (10 μM) were added simultaneously to the suspension. Samples of 10^7^ cells were removed as a function of time and transferred in ice-cold axenic medium containing the same concentration of sorbitol. After 3 washes, cells were resuspended in 1 ml of 40 mM Mes-Na pH 6.5, counted rapidly and lysed by addition of 2 ml of 100 mM NaPi-0.25% (v/v) Triton X-100, pH 10. The fluorescence intensity was measured at λ_ex_ 470 nm and λ_em_ 520 nm. Data were converted into an equivalent volume of internalized fluid using a calibration curve [42].

### Live cell imaging and immunofluorescence

Cells were let to adhere on glass coverslips for 1 h 30 in axenic medium and subjected to stress treatment as indicated. For live cell imaging, cells were observed directly by DIC or fluorescence microscopy. In that latter case, LOFLO nutritive medium (Formedium) was used because of a reduced autofluorescence compared to the original medium. For immunostaining, cells were fixed in axenic medium containing 4% paraformaldehyde (PFA) and 0.1% Triton X-100 for 10 min. After washes in PBS and 1 h incubation in PBS-0.5% BSA, cells were then incubated with the anti-STATc (7H3) antibodies in PBS-0.5% BSA at room temperature for 1 h, washed three times in PBS-0.1% Tween 20 and incubated with Alexa 488-coupled secondary antibodies (Molecular Probes/Life technologies, Saint-Aubin, France) for an additional hour. Actin was stained using Alexa 594-phalloidin (Molecular Probes). Cells were observed directly after three washes in PBS-Tween. Images were acquired on an Axiovert 200 M Zeiss microscope equipped with a Piezo unit. The Axiovision software was used for image acquisition, deconvolution of z-stacks and maximal intensity z-projections.

### Phosphatase treatment

Dephosphorylation assays using the CIP phosphatase (Promega, Charbonnières, France) were performed on SDS denatured samples. An aliquot corresponding to 2 × 10^5^ cells was diluted 20-fold in 50 mM Tris–HCl pH 8.8, 0.66 mM MgSO_4_, 1 mM MgCl_2_, 0.1 mM ZnCl_2_ and 1 mM spermidine. About 15 units of CIP were added and dephosphorylation was performed at 37°C for 30 min. For each time-point, an aliquot receiving no enzyme was treated in parallel as a control. Proteins were precipitated by a 1 h-incubation on ice after the addition of 10% trichloroacetic acid (TCA), centrifuged at 16 000 × *g* and resuspended in denaturing buffer containing 200 mM Tris base. Dephosphorylation was assessed by the abolition of the electrophoretic mobility shift on 8% SDS-PAGE gels.

## Abbreviations

GPCR: G-protein coupled receptor; ART: Arrestin-related trafficking adaptor; Adc: Amoebal arrestin-domain containing protein; Arrdc: Mammalian arrestin-domain containing protein; CIP: Calf intestine phosphatase; PFA: Paraformaldehyde.

## Competing interests

The authors declare no conflict of interests.

## Authors’ contributions

CH, GK and LA designed the study. CH, GK and LA conducted the experiments. CH, GK, TA, JGW and LA drafted the manuscript. All authors read and approved the manuscript.

## Supplementary Material

Additional file 1 **Movie 1.** KAx-3 cells response to 200 mM sorbitol. KAx-3 cells were let to adhere on a glass coverslip in nutritive medium and subjected (t=-30 sec) to 200 mM sorbitol. The movie was taken by time-lapse video microscopy using DIC optics and a 63x objective. The time frame of the movie corresponds to approximately 9 min, with images taken every 10 seconds. Click here for file
